# Decidual derived exosomal miR-99a-5p targets Ppp2r5a to inhibit trophoblast invasion in response to CeO_2_NPs exposure

**DOI:** 10.1186/s12989-023-00524-y

**Published:** 2023-04-20

**Authors:** Hangtian Zhong, Yanqing Geng, Rufei Gao, Jun Chen, Zhuxiu Chen, Xinyi Mu, Yan Zhang, Xuemei Chen, Junlin He

**Affiliations:** 1 School of Public Health, Chongqing Medical University, No.1, Yi Xue Yuan Road, Yuzhong District, Chongqing, 400016 China; 2 Joint International Research Laboratory of Reproduction & Development, Chongqing Medical University, Chongqing, China; 3 College of Pharmacy, Chongqing Medical University, Chongqing, China

**Keywords:** CeO_2_NPs, Placental development, Decidual cells, Trophoblast, Exosome-derived miRNA

## Abstract

**Background:**

The biological effects of cerium dioxide nanoparticles (CeO_2_NPs), a novel material in the biomedical field, have attracted widespread attention. Our previous study confirmed that exposure to CeO_2_NPs during pregnancy led to abnormal trophoblast invasion during early placental development, thereby impairing placental development. The potential mechanisms may be related to low-quality decidualization triggered by CeO_2_NPs exposure, such as an imbalance in trophoblast invasion regulators secreted by decidual cells. However, the intermediate link mediating the “dialogue” between decidual cells and trophoblasts during this process remains unclear. As an important connection between cells, exosomes participate in the “dialogue” between endometrial cells and trophoblasts. Exosomes transfer bioactive microRNA into target cells, which can target and regulate the level of mRNA in target cells.

**Results:**

Here, we constructed a mice primary uterine stromal cell-induced decidualization model in vitro, and detected the effect of CeO_2_NPs exposure on the expression of decidual-derived exosomal miRNAs by high-throughput sequencing. Bioinformatics analysis and dual-luciferase reporter assays were performed to identify target genes of the screened key miRNAs in regulating trophoblast invasion. Finally, the role of the screened miRNAs and their target genes in regulating trophoblast (HTR-8/SVneo cells) invasion was confirmed. The results showed that CeO_2_NPs exposure inhibited trophoblast invasion by promoting miR-99a-5p expression in decidual-derived exosomes, and Ppp2r5a is a potential target gene for miR-99a-5p to inhibit trophoblast invasion.

**Conclusions:**

This study revealed the molecular mechanism by which CeO_2_NPs exposure inhibits trophoblast invasion from the perspective of decidual derived exosomal miRNAs. These results will provide an experimental basis for screening potential therapeutic targets for the negative biological effects of CeO_2_NPs exposure and new ideas for studying the mechanism of damage to trophoblast cells at the decidual-foetal interface by harmful environmental or occupational factors.

**Supplementary Information:**

The online version contains supplementary material available at 10.1186/s12989-023-00524-y.

## Introduction

Nanomaterials are materials with at least one dimension in the three-dimensional structure in the nanometre size (0.1–100 nm) or composed of nano as the basic unit, which have unique physicochemical properties in terms of chemical composition, solubility, surface structure and shape size, and are known as the “new materials of the 21st century” [1, 2]. The rare earth nanomaterial cerium dioxide nanoparticles (CeO_2_NPs) have been studied for their renewable antioxidant capacity in the application of nanomedicines [3]. Therefore, biosafety studies related to CeO_2_NPs need to be supplemented and improved.

The sensitivity of females to environmental factors increases significantly during pregnancy, and the advent of nanomaterials represents a new challenge for the health of pregnant women and the normal development of foetuses. Studies have shown that a variety of nanomaterials damage pregnancy by affecting placental development [4, 5]. The placenta is not only a “kind of a connection between the mother and the foetus”, but it also represents an important selectively permeable barrier for the passage of xenobiotics to the foetus, thus protecting it [6, 7]. And its development involves the normal invasion and differentiation of trophoblast cells. Our previous results showed that CeO_2_NPs exposure during pregnancy led to abnormal trophoblast invasion during early placental development, thereby impairing placental development [8]. The invasion of trophoblast cells is regulated by decidual cells through “dialogue” with each other. Our previous results also found that exposure to CeO_2_NPs during pregnancy resulted in an imbalance of trophoblast invasion regulators secreted by decidual cells, indicating that secreted factors of decidual cells may be involved in the effects of CeO_2_NPs exposure on placental development. However, the intermediate link mediating the “dialogue” between decidual cells and trophoblasts during this process remains unclear.

The role of exosomes as one of the key mediators of intercellular communication has been identified in several physiological and pathological processes. Exosomes are spherical, nanoscale extracellular vesicles, approximately 30–150 nm in diameter [9]. A variety of cells (e.g., immune cells, stem cells, tumour cells, etc.) can release exosomes in a cytosolic manner [10], and exosomes are widely distributed in various body fluids (urine, saliva, blood, amniotic fluid, etc.) [11]. Exosomes are more complex in composition and contain a variety of biomolecules, such as proteins, lipids and nucleic acids (double-stranded DNA and various RNA isoforms) [12]. These molecules are carried by exosomes into body fluids and later taken up by target cells by direct or indirect means, thus regulating gene expression and cellular functions of target cells [13]. It has been reported that bovine endometrial epithelium-derived exosomes contain 254 proteins and 227 miRNAs that are closely associated with the regulation of trophoblast adhesion during embryo implantation [14]. Human decidual-derived exosome contents includes 250 proteins and some miRNAs (miR-21, miR-126, etc.) that play important roles in the regulation of angiogenesis, cell proliferation and invasion [15]. These exosomes can be taken up either autocrinally by ectodermal or stromal cells or in a paracrine manner by epithelial cells or adjacent trophoblast cells [16, 17]. Therefore, decidual-derived exosomes can mediate the regulation of trophoblast invasion and maintain the “offensive-defensive” balance between trophoblasts and decidua during early placental development. Therefore, we hypothesize that CeO_2_NPs exposure may lead to abnormal trophoblast invasion by causing abnormal expression of exosomal genes of decidual origin.

To further reveal the molecular mechanism of abnormal placental trophoblast invasion due to maternal exposure to CeO_2_NPs, we constructed a mouse primary uterine stromal cell-induced decidualization model in vitro and detected the effect of CeO_2_NPs exposure on the expression of decidual-derived exosomal miRNAs by high-throughput sequencing. Bioinformatics analysis and dual-luciferase reporter assays were performed to identify target genes of the screened key miRNAs in regulating trophoblast invasion. Finally, the roles of the screened miRNAs and their target genes in this process were confirmed. These results will help to fully understand the mechanism of biological effects related to CeO_2_NPs exposure, and provide an experimental basis for seeking treatment strategies for the negative biological effects of CeO_2_NPs exposure.

## Results

### Effects of CeO_2_NPs exposure on endometrial stromal cells

**Fig. 1:**
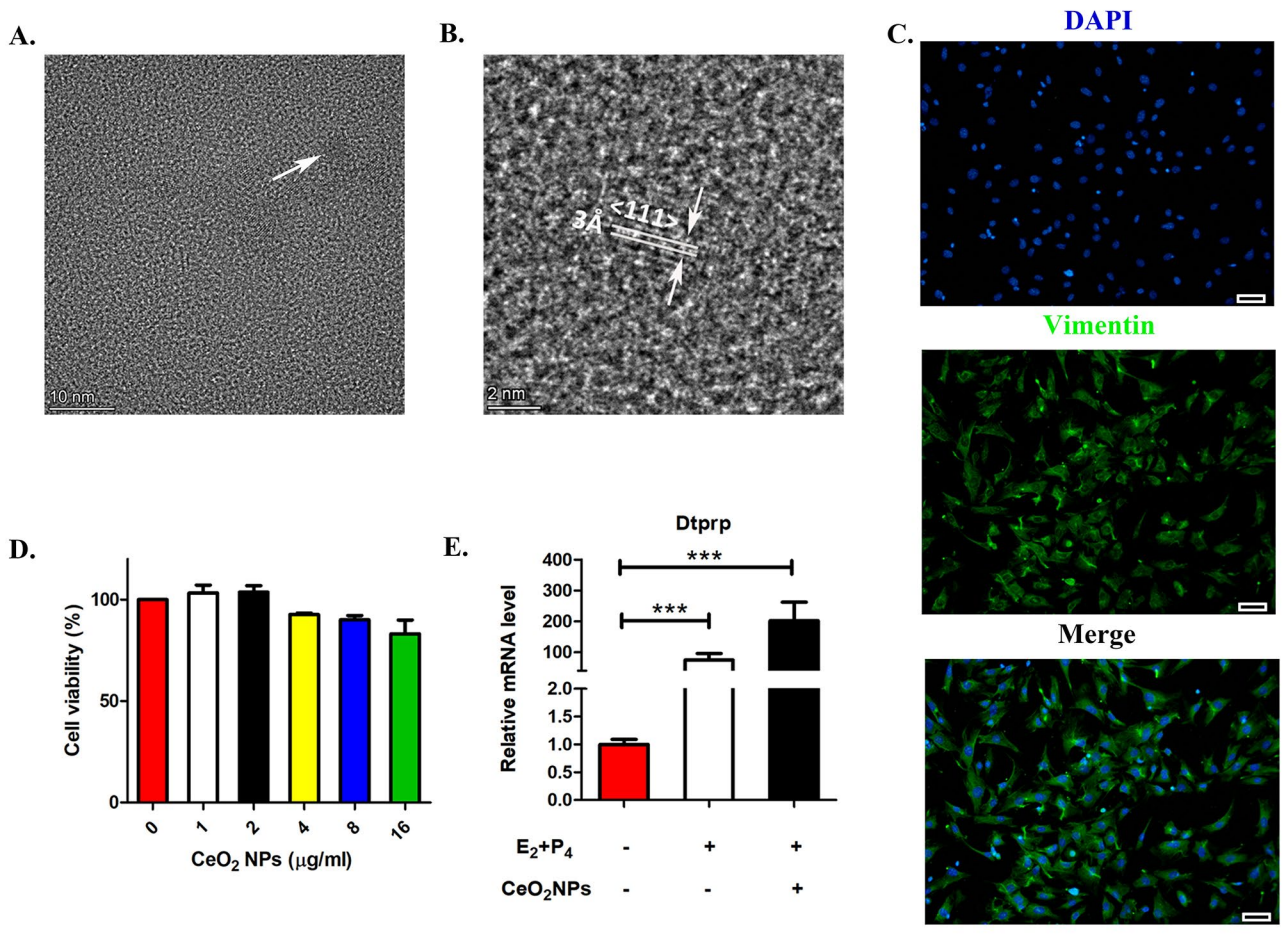
Effects of CeO_2_NPs exposure on endometrial stromal cells. (A) High-resolution FE-TEM images of CeO_2_NPs. (B) Lattice fringe characteristics of CeO_2_NPs. (C) The mESCs were marked by immunofluorescence of Vimentin (scale bar = 50 μm). (D) Cell Counting Kit-8 (CCK8) measurement of cell viability in the mESCs treated with CeO_2_NPs at different concentrations for 24 h. (E) The relative mRNA levels of Dtprp in mESCs after in vitro decidualization induction analysed by RT‒PCR. ***p < 0.001

Field emission transmission electron microscopy (FE-TEM) was used to demonstrate the size and morphology of the prepared CeO_2_NPs. As shown in Fig. 1A, CeO_2_NPs were 3–5 nm in size. The higher resolution images revealed the lattice form of CeO_2_NPs (Fig. 1B), and the lattice fringes with a d spacing of 3 Å and the distance between the (111) lattice planes of CeO_2_ correspond with the previous literature [18].

The immunofluorescence results showed that the isolated stromal cells were positive for Vimentin, indicating the successful isolation of mouse endometrial stromal cells (Fig. 1C). After treatment of primary mouse endometrial stromal cells (mESCs) with CeO_2_NPs at final concentrations of 1 µg/mL, 2 µg/mL, 4 µg/mL, 8 µg/mL and 16 µg/mL respectively for 24 h, the cell counting kit 8(CCK8) results showed that the CeO_2_NPs at different concentrations in each group had no significant effect on mESC viability (Fig. 1D), and subsequent experiments were performed with 16 µg/mL CeO_2_NPs. The mRNA levels of prolactin family 8, subfamily a, member 2(Dtprp), a marker of the endometrial stromal cell decidualization process in mice, were measured by real-time fluorescence quantitative PCR, and the results showed that Dtprp was significantly upregulated in the E_2_- and P_4_-treated cells compared with the controls. In addition, Dtprp was significantly upregulated in the E_2_ and P_4_ combined with CeO_2_NPs exposure group compared with the control group but had no significant changes compared with that of the E_2_-and P_4_-treated group (Fig. 1E). These results suggested that CeO_2_NPs exposure did not affect the decidualization of mESCs in vitro.

### Decidual-derived exosomes could be taken up by HTR-8/SVneo cells

**Fig. 2:**
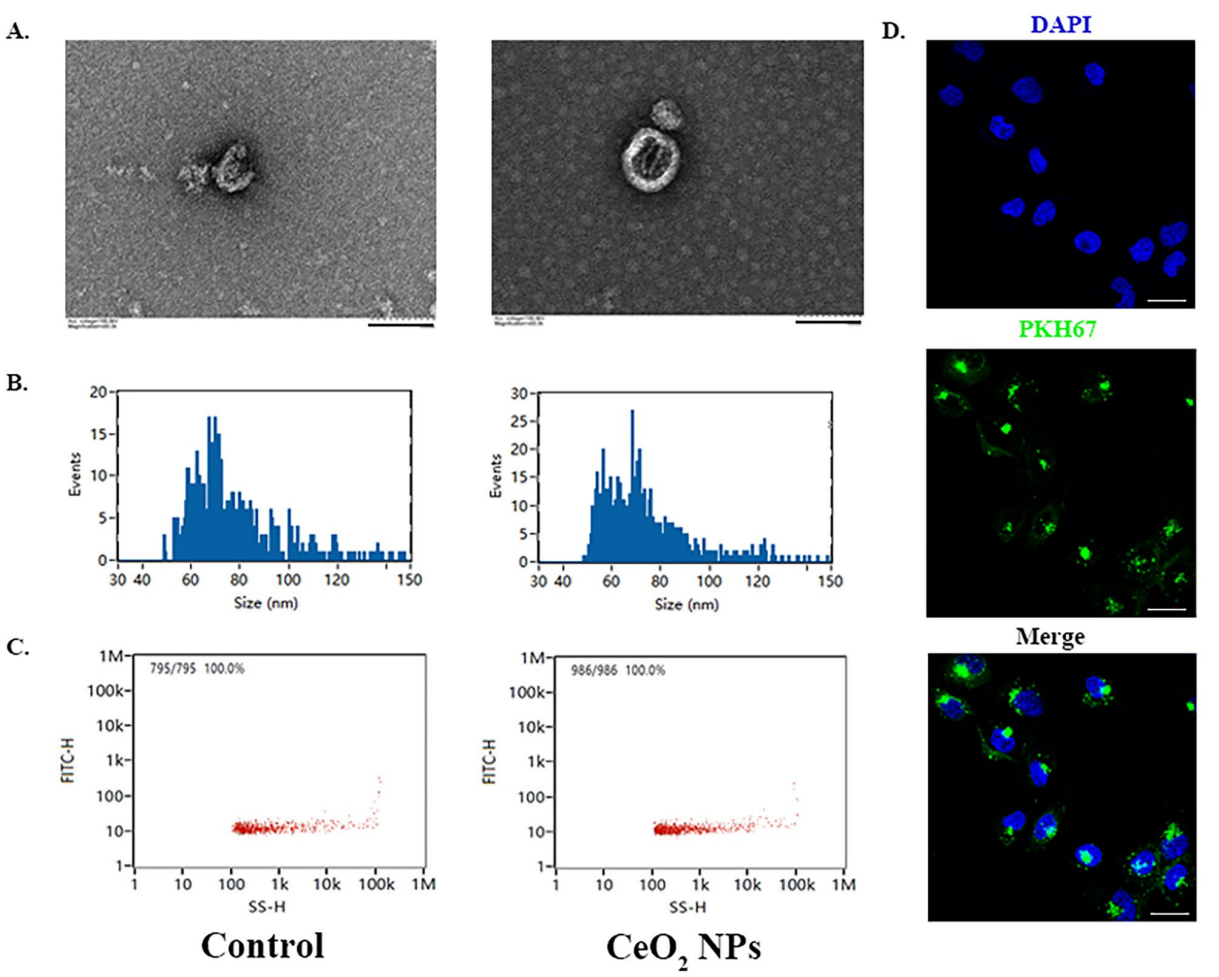
The decidual-derived exosomes could be taken up by HTR-8/SVneo cells. (A) TEM images of exosomes (scale bar = 100 nm). (B) Diameter of exosomes measured by a Flow NanoAnalyzer. (C) Particle concentration of exosomes measured by a Flow NanoAnalyzer. (D) PKH67-labelled exosomes (green) taken up by HTR-8/SVneo cells (blue) (scale bar = 10 μm)

Exosomes were extracted from the culture medium supernatant of decidual cells, and the morphology and particle size of the exosomes were identified. The exosomes from the culture medium supernatant obtained from decidual cells with or without CeO_2_NPs treatment were labelled CeO_2_NPs exosomes and control exosomes, respectively. As shown in Fig. 2A, the transmission electron microscopy results showed an obvious saucer-shaped or hemispherical lipid bilayer typical of an exosomal structure with one side depressed and diameters of approximately 30–150 nm. The average particle size of exosomes in the two groups of exosome samples was determined by a nanoflow detector, and the results showed that the average particle size of the control exosomes was 77.42 nm, and the average particle size of the CeO_2_NPs exosomes was 73.76 nm (Fig. 2B). In addition, the results in Fig. 2C show that the concentration of the control exosomes (particles/mL) was 1.10 × 10^8^ and that of the CeO_2_NPs exosomes (particles/mL) was 1.45 × 10^8^. The above results suggest the successful extraction of exosomes from the cell culture medium supernatant. To clarify that decidual-derived exosomes can be taken up by HTR-8/SVneo target cells and exert certain regulatory effects, we labelled decidual-derived exosomes with PKH67 fluorescent dye, and then the labelled decidual-derived exosomes were cocultured with HTR-8/SVneo cells for 24 h. The results showed that the cytoplasm of HTR-8/SVneo cells appeared with green PKH67 fluorescence, distributed around the DAPI-labelled nucleus (Fig. 2D), suggesting that the decidual-derived exosomes could be successfully taken up by HTR-8/SVneo target cells.

### Exposure to CeO_2_NPs alters the expression of miRNAs in exosomes from decidual cells

**Fig. 3:**
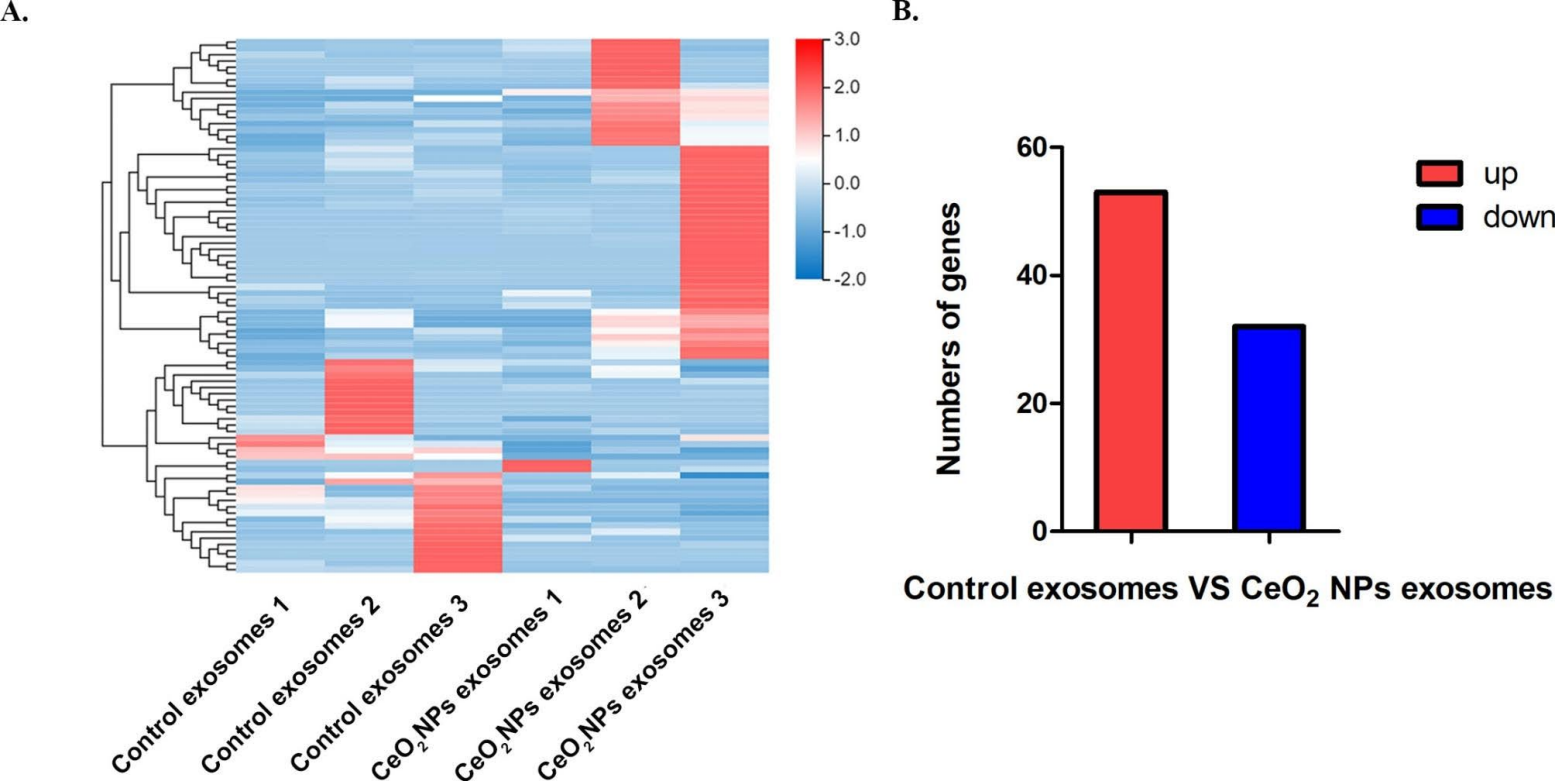
CeO_2_NPs exposure alters miRNA expression in decidual cell-derived exosomes. (A) The heatmap shows differentially expressed miRNAs in exosomes of the two groups. (B) The histogram shows the significantly differentially expressed miRNAs in exosomes between the two groups

To elucidate the effect of CeO_2_NPs exposure on the expression of miRNAs in decidual cell-derived exosomes, we performed small RNA sequencing to analyse the expression profiles of miRNAs in the control exosomes and CeO_2_NPs exosomes. Figure 3 A shows the heatmap of the miRNA expression profiles in the two groups of exosomes and 85 significantly differentially expressed miRNAs (|log2FC| ≥1 and p < 0.01), of which 53 miRNAs were upregulated and 32 miRNAs were downregulated in the CeO_2_NPs exosomes compared to the control exosomes (Fig. 3B).

### Functional clustering and signalling pathway analysis of differentially expressed decidual-derived exosomal miRNAs due to CeO_2_NPs exposure

**Fig. 4:**
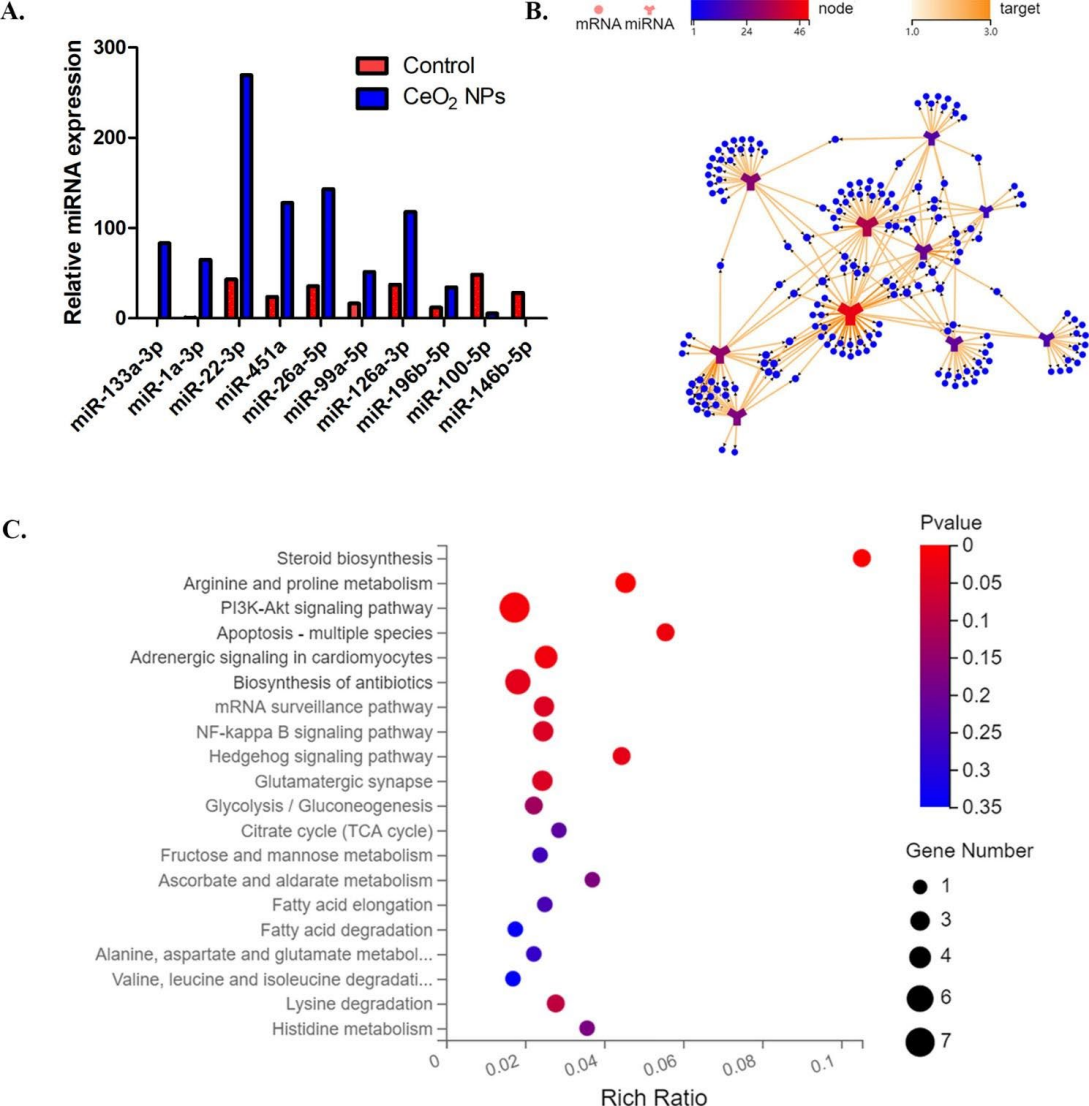
Functional clustering and signalling pathway analysis of differentially expressed decidual-derived exosomal miRNAs due to CeO_2_NPs exposure. (A) Expression levels of 10 miRNAs with large multiple differences in exosomes in the two groups (gained by small RNA sequencing). (B) miRNA target mRNA prediction. (C) KEGG enrichment pathway analysis

Figure 4 A shows 10 of these miRNAs with substantial and significant differences in expression in exosomes, namely, miR-133a-3p, miR-1a-3p, miR-22-3p, miR-451a, miR-26a-5p, miR-99a-5p, miR-126a-3p, miR-196b-5p, miR-100-5p, and miR-146b-5p. The target gene prediction analysis of the 10 significantly different miRNAs was performed using the Dr. Tom system to obtain 169 target genes (Fig. 4B). The KEGG system was used to analyse these 169 target gene-related signalling pathways (Fig. 4C), and the significantly enriched pathways (p < 0.05) were steroid biosynthesis, arginine and proline metabolism, the PI3K-Akt signalling pathway, apoptosis, adrenergic signalling in cardiomyocytes, biosynthesis of antibiotics, and the Hedgehog signalling pathway.

### miR-99a-5p mediated the inhibition of trophoblast invasion after CeO_2_NPs exposure

**Fig. 5:**
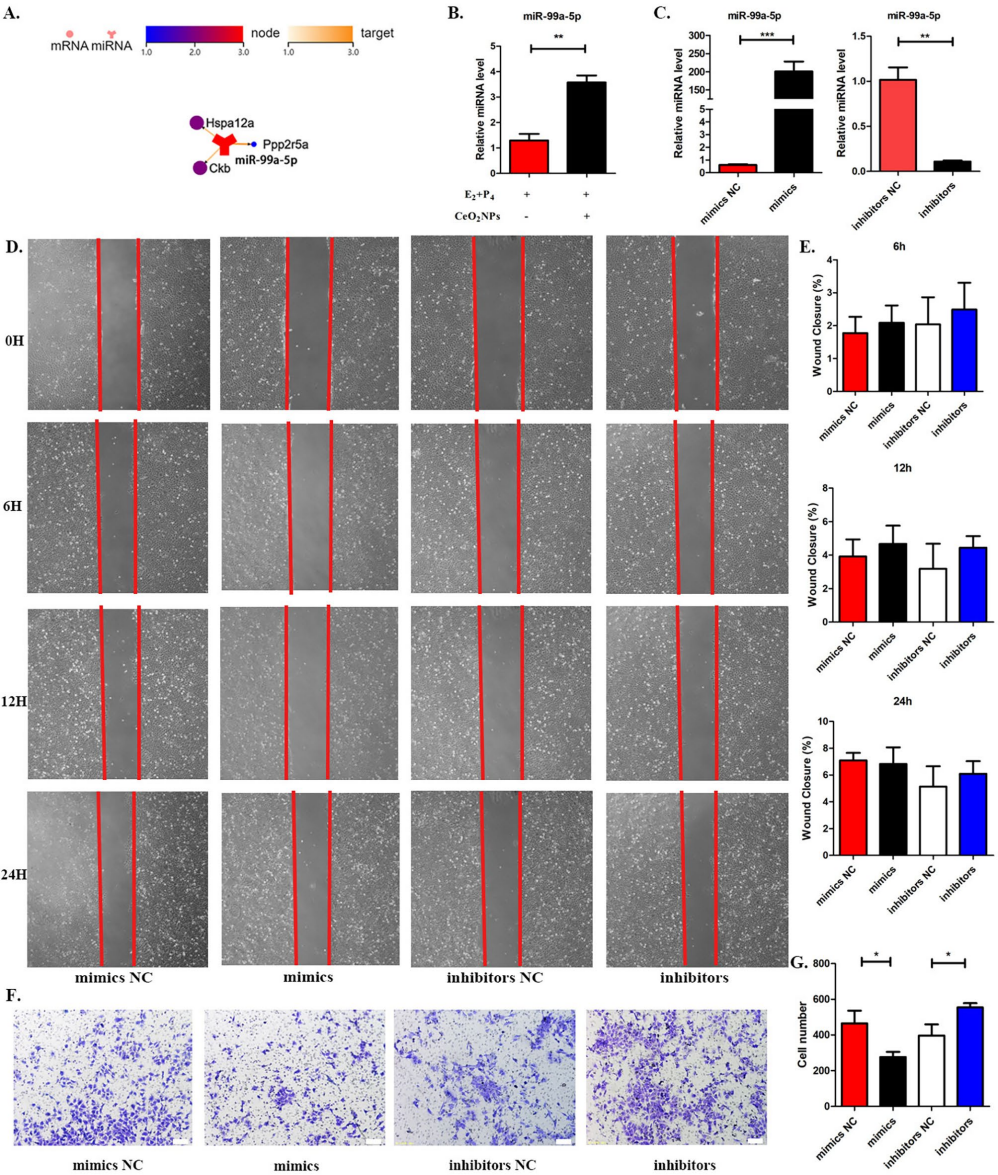
Screening of key miRNAs for expression changes in decidual cells by exposure to CeO_2_NPs. (A) Interaction mapping of miR-99a-5p and its target mRNA. (B) The relative expression levels of miR − 99a-5p in decidual cells detected by RT-PCR. (C) The relative expression levels of miR-99a-5p in HTR-8/SVneo cells. (D) and (E) Cell scratch assay and statistical analysis. (F) and (G) Transwell assay and statistical analysis. *p < 0.05. **p < 0.01. ***p < 0.001

The above significantly enriched pathways contained a total of 17 predicted target genes, and interaction analysis between miR-99a-5p and its target genes was further performed because miR-99a-5p was reported to be involved in the progression of tumour invasion (Fig. 5A). miR-99a-5p was significantly upregulated in the CeO_2_NPs-exposed decidual cell group (Fig. 5B), which was consistent with the exosome sequencing results. Whether miR-99a-5p is involved in the effect of CeO_2_NPs exposure on placental trophoblast invasion is of interest.

HTR-8/SVneo cells were treated with mimics and inhibitors of miR-99a-5p, and quantitative PCR results showed that miR-99a-5p expression was significantly upregulated in the mimics group compared to the mimics NC (negative control) group, suggesting that miR-99a-5p overexpression in HTR-8/SVneo cells was successful (Fig. 5C). miR-99a-5p expression in the inhibitors group was significantly downregulated compared to that in the inhibitors NC group, suggesting that miR-99a-5p expression in HTR-8/SVneo cells was significantly inhibited (Fig. 5C). The results of the scratch assay showed that the healing rate of the HTR-8/SVneo cells with overexpression or inhibition of miR-99a-5p at 6 h, 12 h, and 24 h was not significantly different from that of the control group (Fig. 5D and E), suggesting that miR-99a-5p had no effect on the migration of HTR-8/SVneo cells. Transwell assays showed that the overexpression of the miR-99a-5p group resulted in a significant decrease in the number of cells crossing the chamber, while the number of cells crossing the chamber in the downregulated miR-99a-5p group increased significantly (Fig. 5F and G). The above results suggest that miR-99a-5p inhibits HTR-8/SVneo cells invasion.

### Ppp2r5a was the downstream target gene of miR-99a-5p in HTR-8/SVneo cells

**Fig. 6:**
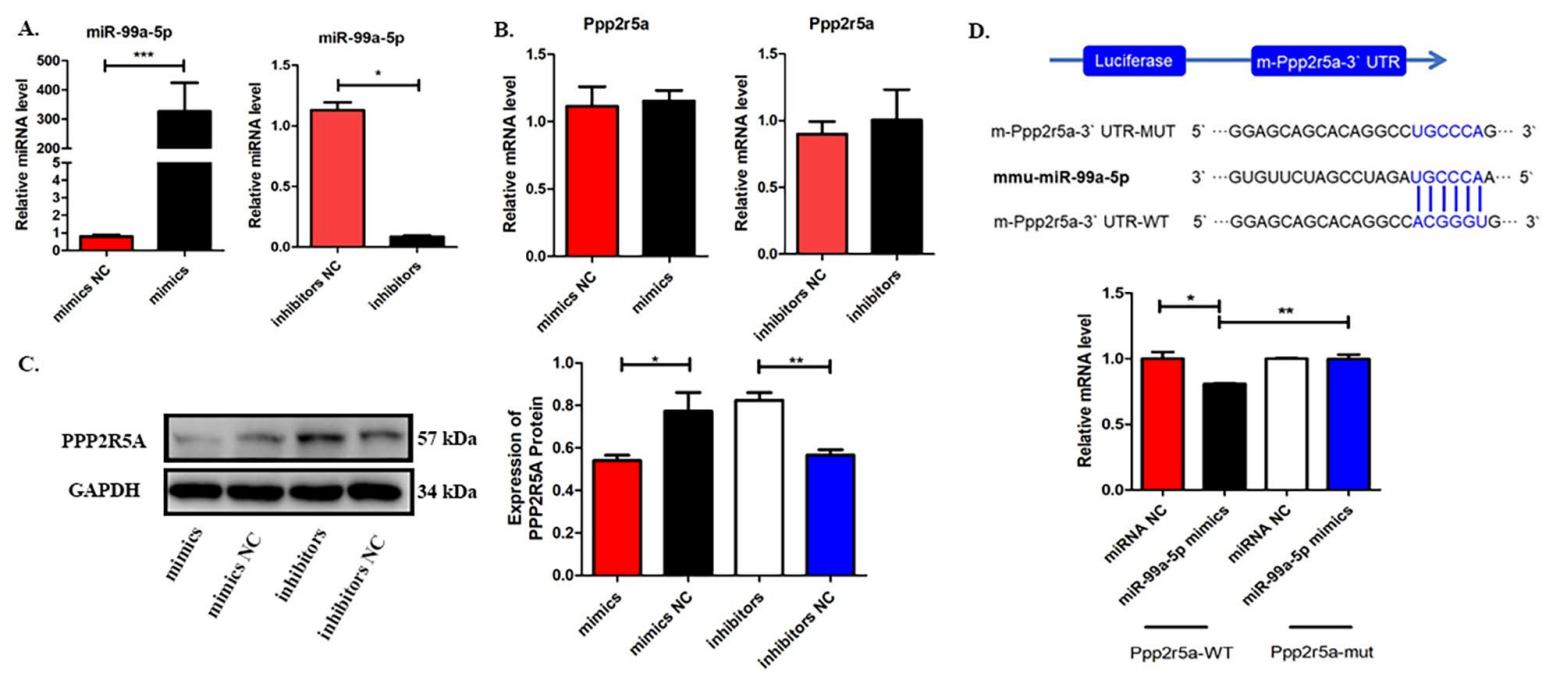
PPP2R5A was the downstream target gene of miR-99a-5p in HTR-8/SVneo cells. (A) The relative expression levels of miR-99a-5p in HTR-8/SVneo cells. (B) Relative expression levels of PPP2R5A in miR-99a-5p-overexpressing and miR-99a-5p-silenced HTR-8/SVneo cells. (C) The expression of PPP2R5A in miR-99a-5p-overexpressing and miR-99a-5p-silenced HTR-8/SVneo cells (left), grey analysis (right). (D) Dual-luciferase reporter assay. *p < 0.05. **p < 0.01. ***p < 0.001

Bioinformatics analysis revealed that the potential target genes of miR-99a-5p include protein phosphatase 2 regulatory subunit B’alpha (Ppp2r5a), creatine kinase B (Ckb) and heat shock protein family A (Hsp70) member 12 A (Hspa12a). PPP2R5A was shown to be involved in the migration and invasion of a variety of tumour cells, suggesting its possible involvement in the inhibition of trophoblast invasion by CeO_2_NPs exposure. HTR-8/SVneo cells were treated with mimics and inhibitors of miR-99a-5p, and quantitative PCR results showed that miR-99a-5p expression was significantly upregulated in the mimics group compared to the mimics NC group, suggesting that miR-99a-5p overexpression in HTR-8/SVneo cells was successful (Fig. 6A). The expression of miR-99a-5p in the inhibitors group was significantly downregulated compared to that in the inhibitors NC group (Fig. 6A), suggesting that the expression of miR-99a-5p in HTR-8/SVneo cells was significantly inhibited. Quantitative PCR results showed that there was no significant change in the mRNA level of Ppp2r5a in HTR-8/SVneo cells after up or downregulation of miR-99a-5p (Fig. 6B). Western blot results showed that the protein expression level of PPP2R5A in the mimics group was significantly lower than that in the mimics NC group, while the protein expression level of PPP2R5A in the inhibitors group was significantly higher than that in the inhibitors NC group (Fig. 6C). Dual luciferase reporter gene analysis revealed that transfection of miR-99a-5p mimics significantly reduced the luciferase activity of m-Ppp2r5a-3`-UTR-WT but had no effect on m-Ppp2r5a-3`-UTR-MUT (Fig. 6D). The above results showed that miR-99a-5p binds to the Ppp2r5a-3`-UTR.

### miR-99a-5p inhibited HTR-8/SVneo cells invasion by targeting Ppp2r5a

**Fig. 7:**
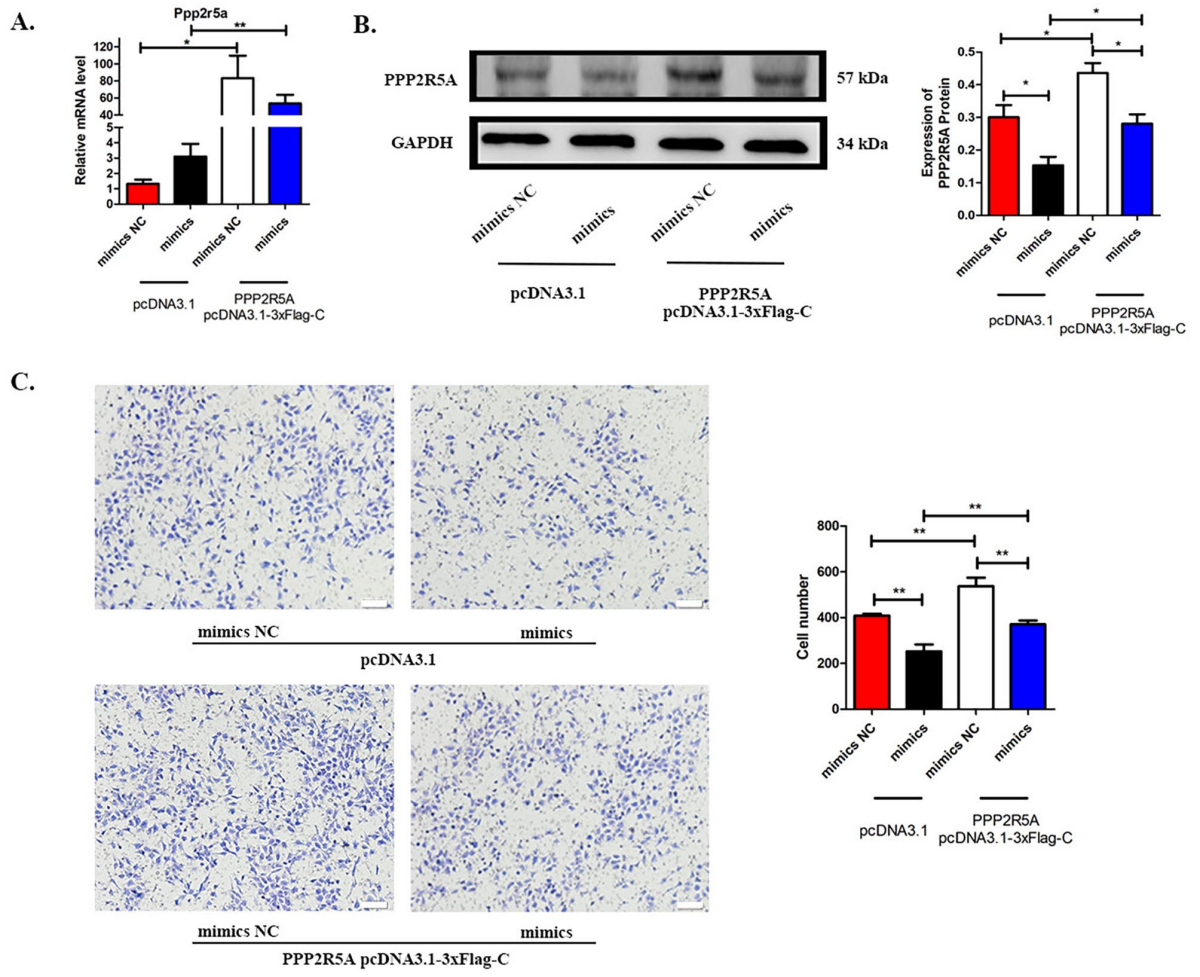
miR-99a-5p inhibited HTR-8/SVneo invasion by downregulating PPP2R5A. (A) The relative expression levels of Ppp2r5a in HTR-8/SVneo cells. (B). The protein expression levels of PPP2R5A in HTR-8/SVneo cells (left), greyscale analysis (right). (C) Transwell assay and statistical analysis. *p < 0.05, **p < 0.01

We found that miR-99a-5p overexpression could downregulate PPP2R5A and result in inhibited invasion of HTR-8/SVneo cells. We also overexpressed Ppp2r5a by plasmid transfection to observe whether the inhibited invasion of HTR-8/SVneo cells caused by miR-99a-5p overexpression could be reversed. As shown in Fig. 7A, overexpression of Ppp2r5a increased the mRNA level of Ppp2r5a in HTR-8/SVneo cells. Co-overexpression of miR-99a-5p and Ppp2r5a reversed the miR-99a-5p-induced decrease in the PPP2R5A protein level (Fig. 7B). In addition, co-overexpression of miR-99a-5p and Ppp2r5a reversed the miR-99a-5p-inhibited invasion of HTR-8/SVneo cells (Fig. 7C). These results confirmed that miR-99a-5p inhibited HTR-8/SVneo cells invasion by downregulating PPP2R5A expression.

## Discussion

The placenta provides nutritional support and immune protection for the foetus, and normal placental development and function are prerequisites for a healthy foetus and a normal pregnancy. Several studies have shown that maternal exposure to some environmental factors (including nanoparticles) during pregnancy can affect the final pregnancy outcome by causing placental damage [19, 20]. Our previous results showed that exposure to CeO_2_NPs during pregnancy can lead to abnormal trophoblast invasion during placental development and impair placental development. Differentiation and invasion of trophoblast cells are necessary to ensure normal placental development. During this process, trophoblast invasion involves complex and elaborate molecular regulatory mechanisms. As cells with secretory functions, decidual cells play an important regulatory role in the invasive migration of trophoblast cells. To further clarify the intermediate link that mediates decidual-trophoblast dialogue, we conducted an in-depth study by constructing an in vitro CeO_2_NPs exposure cell model. The endometrial stromal cells of the mice treated with CeO_2_NPs at final concentrations of 1 µg/mL, 2 µg/mL, 4 µg/mL, 8 µg/mL, and 16 µg/mL for 24 h showed no significant changes in endometrial stromal cell viability, and 16 µg/mL CeO_2_NPs, which had no significant effect on endometrial stromal cell viability in mice, were selected for follow-up studies. Exposure to 16 µg/mL CeO_2_NPs did not inhibit the decidual process of mouse endometrial stromal cells but may lead to changes in the secretory factors of decidual cells. Recent studies have confirmed that NPs can detrimentally affect neighbouring cells in a manner similar to the radiation-induced bystander effect in addition to affecting the health of directly exposed cells [21]. There was a trend towards increased levels of the decidualization marker Dtprp compared to that of the CeO_2_NPs nonexposed group, which can inhibit excessive invasion of trophoblast cells into the uterine decidua. This finding is consistent with our previous findings that CeO_2_NPs exposure can result in an imbalance of the pro- and antitrophoblast invasion factors secreted by decidual cells, resulting in abnormal trophoblast invasion and ultimately impaired placental development. The interactions between CeO_2_NPs and the cell membrane, whether CeO_2_NPs are internalized into stromal cells and the changes in signalling pathways in decidual cells should be examined in future studies. On the other hand, the use of murine stromal cells, instead of the human ones, is probably a limit of the study since the human endometrial stromal cells would have been more appropriate for drawing conclusions about the effect of these exotic nanomaterials on human health in pregnancy.

In vitro models allow to evaluate the effect on one compartment or on the other (e.g. trophoblast cells [22]; embryonic stem cells [23]; endometrial cells). These nice models allow to study the link mediating the so complex and highly regulated blastocyst-endometrium crosstalk, encompassing both decidual and trophoblast compartments. Thus, the in vitro cell model plays a key role in evaluating the potential toxic effects of nanoparticles in the environment. As one of the important mediators that mediate cellular dialogue, exosomes can be involved in a variety of intercellular communications. Human decidual-derived exosome contents include 250 proteins and some miRNAs (miR-21, miR-126, etc.) that play important roles in the regulation of angiogenesis, cell proliferation and invasion. Several studies have shown that exosomes play an important regulatory role in female pregnancy. During a normal healthy pregnancy, the number of exosomes present in maternal plasma increases significantly during the first trimester along with increasing gestational age, with placenta-derived exosomes (e.g., PLAP + exosomes) [19]. During embryo implantation, blastocysts can release exosomes, thus establishing “communication” with endometrial epithelial cells, and blastocyst-derived exosomes can be internalized by endometrial epithelial cells to receive signals, creating conditions for the establishment of a receptive state of the uterus and implantation of blastocysts [20]. We isolated mouse endometrial stromal cells in vitro, induced their decidualization in the presence of oestrogen and progestin, and established an in vitro decidual cell CeO_2_NPs exposure model to obtain exosomes from the culture medium supernatant of decidual cells, and the results of exosome identification confirmed that decidual cells could indeed release exosomes. In the exosome extraction assay, we found that decidual-origin exosomes were not abundant, so it was difficult to directly observe the effect of decidual-origin exosomes on trophoblast migration or invasion. However, the exosome uptake assay confirmed that PKH67-labelled decidual-derived exosomes could be successfully taken up by HTR-8/SVneo cells, indicating that trophoblast cells could be regulated by the information molecules carried in decidual-derived exosomes.

Among the various biomolecules contained in exosomes, miRNAs are the most intensively studied. miRNAs are a small noncoding single-stranded RNA molecules of approximately 21 nucleotides in length encoded by endogenous genes that degrade mRNA at the transcriptional level or inhibit its translation into proteins after transcription by binding to the 3`-UTR of mRNA with complete or partial base complementary pairing and thus exerting some regulation on downstream target genes [24]. These molecules are highly conserved among species and play important roles in angiogenesis, cell proliferation and invasion by regulating the expression of downstream target genes. The miRNA sequencing and bioinformatics analysis of decidual-derived exosomes revealed that there were 85 significantly differentially expressed miRNAs in the decidual-derived exosomes of the CeO_2_NPs-exposed group compared with the decidual-derived exosomes of the control group, 10 of which were expressed in higher amounts in the exosomes, and the miRNAs with higher fold change ranking were miR-133a-3p, miR-1a-3p, miR-22-3p, miR-451a, miR-26a-5p, miR-99a-5p, miR-126a-3p, miR-196b-5p, miR-100-5p and miR-146b-5p. A total of 169 predicted downstream target genes were obtained by the Dr.Tom system of BGI. After functional analysis of the predicted downstream target genes, it was found that the significantly enriched pathways were as follows: steroid biosynthesis pathway, arginine and proline metabolism pathway, the PI3K-Akt signalling pathway, apoptosis, adrenergic signalling in cardiomyocytes, biosynthesis of antibiotics and the Hedgehog signalling pathway. These significantly enriched pathways contain a total of 17 target genes. Through the interaction analysis of these 17 target genes and the 10 screened miRNAs, 6 miRNAs and their 10 target genes were further screened. The target genes of miR-22-3p, miR-99a-5p, miR-100-5p and miR-126 A-3p were mainly concentrated in steroid biosynthesis pathways, arginine and proline metabolism, and the PI3K-Akt signalling pathway which are related to placental development and pregnancy.

Based on the above study, miR-99a-5p was significantly upregulated in the CeO_2_NPs-exposed decidual group, and this result was consistent with the exosome sequencing results. The general term for miR-99a-5p is miR-99a. Murine-derived miR-99a is mainly localized on chromosome 16, and its Gene Ontology (GO) classification shows that it is mainly located in the biological process and cellular component categories and plays a role in cell differentiation, cell population proliferation, and immune system processes [25]. miR-99a is highly expressed in the embryonic ectoderm, digestive system, cardiovascular system, musculoskeletal system and reproductive system. Studies have shown that miR-99a-5p acts as a tumour suppressor and exerts inhibitory effects in several tumour cells, such as in human oral lymphoid carcinoma cells TSCC1, and miR-99a-5p can affect the proliferation, migration and invasion of human oral cancer cells by inhibiting NOX4 expression [26]. In breast cancer cell lines (MCF-7 and MDA-MB-231), miR-99a-5p significantly inhibited the proliferation and invasion of breast cancer cells and promoted apoptosis by targeting CDC25A and inhibiting its expression in cells [27]. In bladder cancer cells, miR-99a-5p enhanced RAD001-induced apoptosis in human bladder uroepithelial carcinoma cells (5637 and T24 cells) by targeting mTOR as a tumour suppressor [28]. In addition, miR-99a-5p significantly inhibited the proliferation, migration and invasion of human aortic smooth muscle cells (ASMCs) by targeting HOXA1 to attenuate the formation of atherosclerotic lesions in the course of atherosclerotic disease [29]. To date, the effects of miR-99a-5p on trophoblast cells and related mechanisms have not been reported. We found that miR-99a-5p could inhibit trophoblast invasion, and its downstream potential target gene, protein phosphatase 2 regulatory subunit B’alpha (Ppp2r5a), was obtained by bioinformatics prediction. Our results confirmed that PPP2R5A is the target gene of miR-99a-5p in HTR-8/SVneo cells. Co-overexpression of miR-99a-5p and Ppp2r5a reversed only the miR-99a-5p-inhibited invasion of HTR-8/SVneo cells. These results confirmed that miR-99a-5p inhibited HTR-8/SVneo cells invasion by downregulating PPP2R5A expression. PPP2R5A is one of the regulatory subunits of protein phosphatase 2 A (PP2A) and plays an important role in regulating cancer development. PPP2R5A participates in a variety of tumour related signalling pathways [30]. PPP2R5A can activate the MAPK pathway, while the MAPK pathway is involved in promoting breast cancer cell invasion [31] and non-small cell lung cancer cell invasion [32]. PPP2R5A can also promote P53 degradation, while in pancreatic cancer cells, P53 degradation can promote cell invasion [33]. The role of PPP2R5A in trophoblast cells also needs further investigation.

## Conclusion

**Fig. 8:**
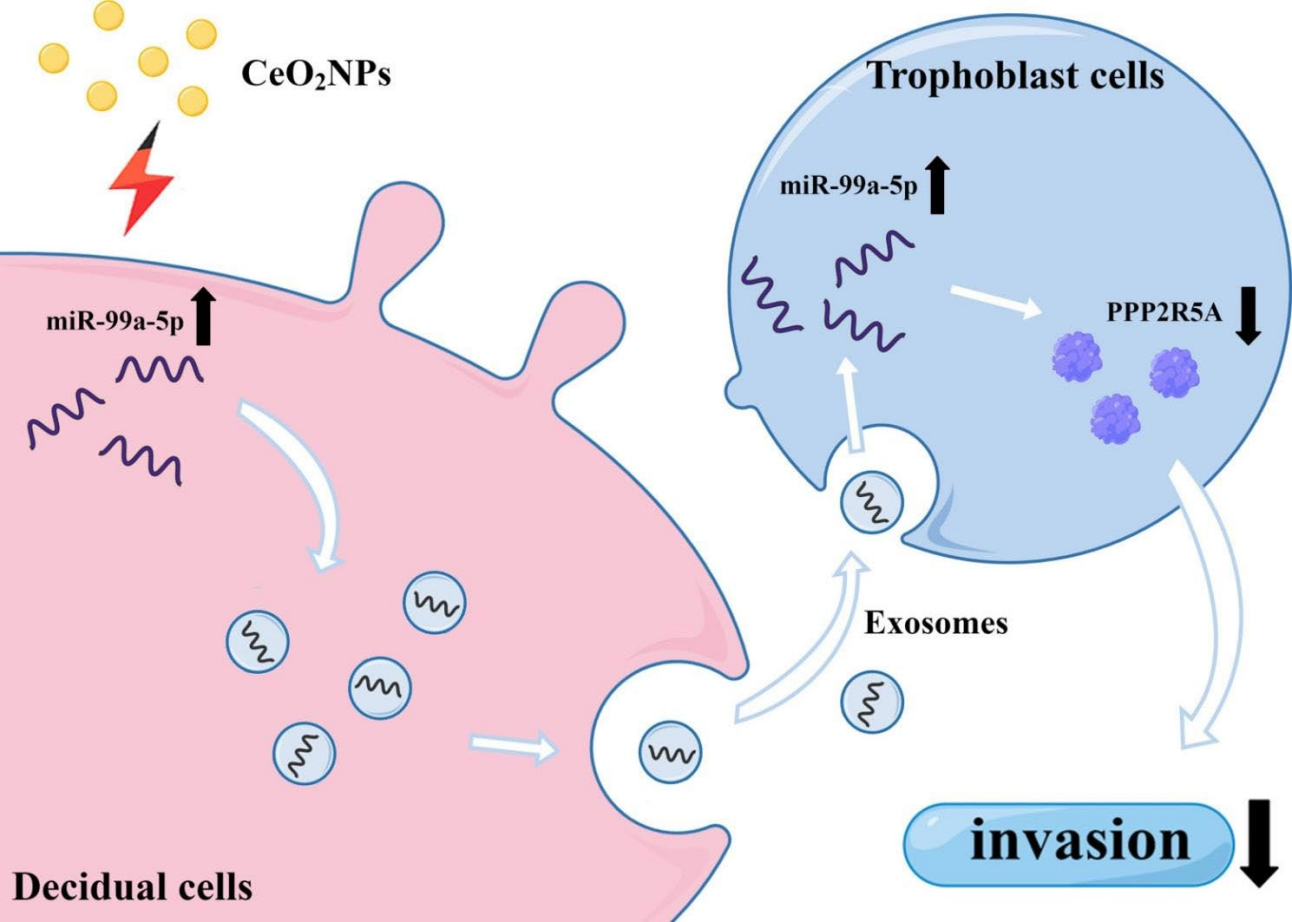
Diagram of the mechanism of action of CeO_2_NPs exposure during pregnancy leading to impaired trophoblast invasion by altering the genetic composition of decidual-derived exosomes. CeO_2_NPs exposure can inhibit trophoblast invasion by upregulating miR-99a-5p expression in decidual -derived exosomes and downregulating PPP2R5A expression therein after uptake by trophoblast cells

In summary, we used the decidual-derived secretory factor involved in mediating trophoblast invasion as the entry point, and constructed a CeO_2_NPs-exposed decidual cell model with the help of stromal cell induction of decidualization in vitro to analyse the effect of CeO_2_NPs exposure on the expression of decidual-derived exosomal miRNAs and to reveal the molecular mechanism by which CeO_2_NPs exposure during pregnancy impairs placental development. The results showed that CeO_2_NPs exposure inhibited trophoblast invasion by upregulating the expression of miR-99a-5p in decidual-derived exosomes and downregulating the expression of PPP2R5A after uptake by trophoblast cells (Fig. 8). The above results will provide an experimental basis for screening targets for the negative effects of CeO_2_NPs.

## Materials and methods

### Nanoparticles and characterization

The CeO_2_NPs were prepared by a microemulsion process as described in our previous works [8]. Briefly, 5 mL of CeO_2_NPs in toluene phase (0.8 mg mL^− 1^) were added 1 mL of ammonia solution and 50 mL of anhydrous ethanol to wash one time and then added 25 mL pure water to wash three times. Finally, the CeO_2_NPs precipitate was dissolved in 4 mL of the corresponding solvent. The concentration of pure CeO_2_NPs was 1 mg mL^− 1^. Field emission transmission electron microscopy (FEI Tecnai G2 F20) was used to determine the size and morphology of the CeO_2_NPs. All reagents used for CeO_2_NPs synthesis were obtained from Sigma‒Aldrich (St. Louis, USA, www.sigmaaldrich.com) and were of analytical grade.

### Animal treatment

Female BALB/c mice were purchased from Beijing Vital River Laboratory Animal Technology Co., Ltd (Certificate No.: SCXK (JING) 20,160,006). The mice were housed in the specific pathogen-free facility of Chongqing Medical University under a normal light–dark cycle (12:12) at 22 ± 2 ℃ and 50% humidity with access to a standard chow diet and water. primary mouse endometrial stromal cells (mESCs) were isolated from the mice. All experiments were approved by the Chongqing Medical University Animal Care and Use Committee.

### Cell culture and treatment

#### Isolation and identification of primary mESCs

mESCs were isolated as previously described [34]. In brief, uteri were isolated from sexually mature female mice and cut into several small tissues (3–4 mm^3^). First, the tissues were digested with enzyme I (1 U/mL of trypsin (Sigma‒Aldrich, Cat. No. T4799-5G) and 1 U/mL of dispase II (Roche, Cat. No. 04942078001)) at 4 °C for 1 h, room temperature for 1 h and finally 37 ℃ for 1 h. Second, the digested tissues were vigorously blown by pipette tips to make them sticky. Third, after centrifugation, the remaining tissues were incubated with 1 U/mL of collagenase (Gbico, Cat. No. 17101-105) at 37 °C for 30 min and then shaken vigorously every 10 min until the supernatants became turbid. Then, the supernatant was passed through a 70-µm nylon filter to eliminate clumps of epithelial cells, followed by centrifugation. The cell pellets were washed twice with HBSS and suspended in 10% complete medium consisting of Dulbecco’s modified Eagle’s medium/Nutrient Mixture F-12 Ham medium (DMEM-F12, Sigma‒Aldrich, Cat. No. D6434-500ML) supplemented with 10% charcoal-stripped foetal bovine serum (CS-FBS, BI, Cat. No. 04-201-1 A), 100 U/mL penicillin (Beyotime, Cat. No. C0222) and 100 mg/mL streptomycin (Beyotime, Cat. No. C0222). The suspended cells were plated. After an initial culture for 60 min, the isolated stromal cells were further cultured in 20% complete medium consisting of DMEM-F12 supplemented with 20% CS-FBS, 100 U/mL penicillin and 100 mg/mL streptomycin at 37 °C and 5% CO_2_ and identified. Cell identification was performed by immunofluorescence detection of Vimentin.

#### mESC treatment with CeO_2_NPs

Based on the exposure dose of 4 µg/mL CeO_2_NPs in HeLa cells studied by Yang et al [35], CeO_2_NPs were added to primary mESCs and cultured in vitro for 24 h at final concentrations of 1 µg/mL, 2 µg/mL, 4 µg/mL, 8 µg/mL, and 16 µg/mL for CCK-8 assay.

#### Decidualization of mESCs induced in vitro

According to the related reference [34], the mESCs and CeO_2_NPs-exposed mESCs group were cultured with 2.5% complete medium consisting of DMEM-F12 supplemented with 2.5% CS-FBS, 100 U/mL penicillin and 100 mg/mL streptomycin, and then E_2_ (10 nM) and P_4_ (1 µM) were added to the medium to induce decidualization for 72 h, the CeO_2_NPs-exposed mESCs group were also added 16 µg/mL of CeO_2_NPs for 72 h. The decidual cell culture medium was recovered and frozen at -80 ℃ for storage.

#### HTR-8/SVneo cell transfection

The HTR-8/SVneo cell line was obtained from MeisenCTCC (MeiSen, CTCC-400-0143) and was cultured with 10% foetus bovine serum RPMI-1640 medium (Sigma-Aldrich, Cat. No. R8758-500ML) at 37 °C and 5% CO_2_. The cell transfection experiments were carried out according to the instructions. Mimics (chemically synthesized mature miRNA-99a-5p double chain, to overexpression miRNA-99a-5p)/mimics NC (negative control), inhibitors (chemically modified mature miRNA-99a-5p complementary single chain, to inhibit miRNA-99a-5p function)/inhibitors NC sequences, control plasmid (pcDNA3.1) and Ppp2r5a overexpression plasmid (PPP2R5A pcDNA3.1-3xFlag-C) were designed by Guangzhou Ruibo Biological Co., Ltd. The steps were as follows: when the cells grew to approximately 60-80%, 50 nM of mimics and mimics NC, 100 nM of inhibitors and inhibitors NC, 2 µg of control plasmid and Ppp2r5a over expression plasmid were transfected with 2.5-4 µL of Lipofectamine-2000 (Invitrogen, Cat. No. CN2478853) for 6 h, then the transfected cells were cultured in replaced 10% foetus bovine serum RPMI-1640 medium at 37 °C, 5% CO2 condition for 24 h and then the transfected cells were treated for subsequent experiments.

### Cytotoxicity assay (CCK-8 assay)

The CeO_2_NPs were added to primary mESCs and cultured in vitro at final concentrations of 1 µg/mL, 2 µg/mL, 4 µg/mL, 8 µg/mL and 16 µg/mL, and 10 µL of CCK-8 solution was added to each well after 24 h of incubation. The culture plates were incubated in an incubator at 37 ℃ for 1–4 h and the absorbance at 450 nm was measured by a microplate reader.

### Isolation and identification of exosomes and miRNA sequencing

Exosomes were isolated using Exo-spin™ (Cat. No. EX01), an exosome extraction kit provided by Shanghai Xiaopeng Biological Co. Electron microscopy identification, particle size identification, miRNA sequencing and bioinformatics analysis of purified exosomes were completed by Shenzhen BGI Co., Ltd.

### Immunofluorescence analysis

The cells were fixed with 4% paraformaldehyde at room temperature for 10 min and then permeabilized with 0.1% Triton at room temperature for 10 min. Then the cells were incubated with 5% BSA at 37 °C for 1 h. After that, the cells were incubated overnight at 4 °C with the corresponding primary antibody dilution (Vimentin, 1;100, Rabbit, Cell Signalling).Then, the cells were incubated with the corresponding fluorescent secondary antibody dilution at 37 °C for 1 h. After that, the cells were incubated with DAPI for 10 min at room temperature. Finally, the crawl slices were sealed with antifluorescent bursting solution, and pictures were taken for fluorescence observation.

### Real-time PCR

Total RNA was extracted from the cells using RNAiso Plus Reagent (Sigma-Aldrich, Cat. No. T9424-200ML) according to the manufacturer’s protocol. Reverse transcription was conducted using the Prime Script™ RT reagent Kit (TaKaRa, Cat. No. RR036A) and Shanghai Sangon miRNA first strand cDNA synthesis (tailing method) kit (Cat. No. B532451). The cDNA was amplified using gene-specific primers and TB Green (Takara, Cat. No. RR820A and Shanghai Sangon, Cat. No. B532461), and PCR was performed by Bio-Rad CFX Manager 3.1 Detection System. Experiments were performed in triplicate for each sample, and the 2^−ΔΔCt^ method was used to calculate the relative gene expression in different tissue samples with β-actin, Gapdh and U6 as the internal controls. The specific primers for qPCR were designed and synthetized by Sangon Biotech Co., Ltd., and the sequences of the primers for qPCR are shown in Table S1.

### PKH67-labelled exosome uptake by HTR-8/SVneo cells

First, the PKH-67 dye was added to the exosomes, mixed for 1 min, and then incubated for 10 min, followed by the addition of 500 µL of PBS and mixing. Next, exosomes were extracted again according to the exosome extraction method to remove excess dye. Next, the stained exosomes were added to HTR-8/SVneo cells preseeded on the crawler at 37 °C for 12 h. HTR-8/SVneo cells on the crawler were then fixed in 4% paraformaldehyde for 10 min at room temperature and incubated with DAPI for 10 min. Finally, the crawls were sealed with antifluorescence burst solution and photographed for fluorescence observation.

### Cell scratch assay

HTR-8/SVneo cells were inoculated in 24-well plates at an appropriate density, and after transfection treatment, when the cell density reached 90% or more, the cells in the 24-well plates were automatically scratched with a fully automated well plate cell scratcher, followed by washing off the scratched cells with PBS, adding serum-free medium, putting them into the cell culture incubator, and observing the change in scratch area by taking pictures under an inverted microscope at 0, 6, 12 and 24 h to evaluate the cell migration.

### Transwell assay

The Matrigel Matrix (BD, Cat. No. 356,234) was first diluted 1:10, and then added into Transwell. The Matrigel Matrix could be wrapped around the upper surface of the bottom membrane of the Transwell, and placed at 37 °C for 60 min to polymerize the Matrigel Matrix. Then the transfected HTR-8/SVneo cells (approximately 4 × 10^4^) were inoculated with 200 µL of serum-free medium and added to the Transwell. Five hundred microlitres of medium containing 20% foetus bovine serum was generally added to the lower chamber of the 24-well plate and incubated in a cell incubator for 24 h. Finally, the Transwell was removed, and the cells were fixed in ice-cold methanol at 4 °C for 20 min. The cells were then stained with crystal violet for 40 min, the upper Matrigel Matrix and the cells that did not cross the Matrigel Matrix were gently wiped off with a cotton swab, and the number of cells that crossed the Matrigel Matrix was counted under a light microscope to assess the invasive ability of the cells.

### Western blot analysis

Total protein was extracted from uterine decidual tissue samples using cell lysis buffer (Beyotime, Cat. No. P0013F). Samples were separated by 10% SDS–PAGE and transferred to PVDF membranes. The membranes were blocked in 5% w/v nonfat milk for 90 min and then incubated overnight at 4 ℃ with primary antibodies (PPP2R5A (1:500, Rabbit, Polyclonal, Cat. No. DF4482), GAPDH (1:3000, Mice, Monoclonal, Cat. No. T0004), Affinity Bioscience). Then, all membranes were incubated with the corresponding secondary antibody (Boster, Cat. No. BST17D11B17E50) at 37 °C for 60 min. Band intensities were measured using enhanced chemiluminescence reagents (NCM Biotech, Cat. No. P10300) and quantified via densitometry using Quantity One version 4.4.0 software.

### Dual-luciferase reporter assay

The potential target sites of mmu-miR-99a-5p in the 3`-UTR of the Ppp2r5a mRNA sequence were predicted by the starBase databases (https://starbase.sysu.edu.cn/). The 3`-UTR fragments from Ppp2r5a mRNA containing the predicted mmu-miR-99a-5p binding site were cloned into PGK-hluc/MCS-Luc constructs at the XhoI and NotI sites. All of the sequences of the target gene was shown in Table S1. Then, 293T cell transfection was performed with 100 µL of Opti-MEM medium, 2 µg corresponding plasmid, 5 µL of 20 µM corresponding miRNA and 15 µL of Lipofectamine 2000. Then the expression of luciferase between the groups was detected by the Dual-Luciferase Reporter Assay System (E1910, Promega) after transfection for 30 h.

### Statistical analysis

Prism 5.0 (GraphPad Software) was employed for statistical analyses. All the data are based on at least three replications and are shown as the mean ± standard deviation (SD). Unpaired t tests or one-way ANOVA were performed for statistical analysis, and the differences were considered significant at *p < 0.05, **p < 0.01, and ***p < 0.001.

## Electronic supplementary material

Below is the link to the electronic supplementary material.

Supplementary Material 1

## Data Availability

All the original data are available upon reasonable request for correspondence authors.

## List of abbreviations

CeO_2_NPs: Cerium dioxide nanoparticles
FE-TEM: Field emission transmission electron microscopy
CCK-8: Cell Counting Kit-8
HTR-8/SVneo: Human chorionic trophoblast cell line
Ppp2r5a: Protein phosphatase 2 regulatory subunit B’alpha
Ckb: Creatine kinase B
Hspa12a: Heat shock protein family A member 12 A
GO: Gene Ontology
mESCs: mouse endometrial stromal cells
DMEM-F12: Dulbecco’s modified Eagle’s medium/Nutrient Mixture F-12 Ham medium
CS-FBS: charcoal-stripped foetus bovine serum
NC: negative control
SD: standard deviation
PBS: phosphate buffered saline
PBST: tris-buffered-saline with tween
PVDF: polyvinylidene fluoride
mM: millimolar
µM: micromolar
µl: microliter
h: hour
KDa: kilodalton.
