# Supplementary material for: Decidual derived exosomal miR-99a-5p targets Ppp2r5a to inhibit trophoblast invasion in response to CeO2NPs exposure
Source: Part Fibre Toxicol. 2023 Apr 20;20:14. doi: 10.1186/s12989-023-00524-y (PMC10116836; doi:10.1186/s12989-023-00524-y)
Supplement: Supplementary file 1 — Supplementary Material 1 [file 12989_2023_524_MOESM1_ESM.docx]

**Decidual derived exosomal miR-99a-5p targets Ppp2r5a to inhibit trophoblast invasion in response to CeO_2_NPs exposure**

Hangtian Zhong^1,2^, Yanqing Geng^2^, Rufei Gao^1,2^, Jun Chen^3^, Zhuxiu Chen^1,2^, Xinyi Mu^2^, Yan Zhang^1,2^, Xuemei Chen^1,2^, Junlin He^1,2^*

^1^ School of Public Health, Chongqing Medical University, Chongqing, China

^2^ Joint International Research Laboratory of Reproduction & Development, Chongqing Medical University, Chongqing, China

^3^ College of Pharmacy, Chongqing Medical University, Chongqing, China

Yanqing Geng and Hangtian Zhong contributed equally to this work and should be considered co-first authors.

*Corresponding author: Prof. Junlin He

E-mail: [hejunlin@cqmu.edu.cn](mailto:hejunlin@cqmu.edu.cn)

Full address: Box 197#, No.1, Yi Xue Yuan Road, Yuzhong District, Chongqing Medical University, Chongqing 400016, China.

**Table S1** **Primer sequences used in this study**

| Gene name | Sequences of primers(5' - 3') |
| --- | --- |
| Dtprp | Forward：TACCCACGTAAGGTCATC |
|  | Reverse：CTCAGAGCCAGAAATCA |
| Hspa12a | Forward：CAGCCTCTATCTACTGCCGAAAGC |
|  | Reverse：TGCCGATTACGCCGTATGTGTTC |
| Ppp2r5a | Forward：GCCAATTATGTTTGCCAGTTTG |
|  | Reverse：CGAAAAGCTTGCCATTCATTTC |
| Ckb | Forward：CGACTTCAGAAGCGAGGCACAG |
|  | Reverse：TCACTCCGTCCACCACCATCTG |
| Gapdh | Forward：CAGGAGGCATTGCTGATGAT |
|  | Reverse：GAAGGCTGGGGCTCATTT |
| β-actin | Forward：CTCTCCCTCACGCCATC |
|  | Reverse：ACGCACGATTTCCCTCTC |
| U6 | Forward：GCTTCGGCAGCACATATACTAAAAT |
|  | Reverse：CGCTTCACGAATTTGCGTGTCAT |
| miR-99a-5p | Forward：CAACCCGTAGATCCGATCTTGTG |
| miR-100-5p | Forward：CAACCCGTAGATCCGAACTTGTG |
| miR-22-3p | Forward：CAAGCTGCCAGTTGAAGAACTGT |
| miR-126a-3p | Forward：GCTCGTACCGTGAGTAATAATGCG |
| m-Ppp2r5a-3` UTR-MUT | ···GGAGCAGCACAGGCCUGCCCAG··· |
| m-Ppp2r5a-3` UTR-WT | ···GGAGCAGCACAGGCCACGGGUG··· |
